# Preserving joint healthspan in knee osteoarthritis: a geroscience-guided review of non-surgical therapies and genicular artery embolization

**DOI:** 10.1007/s11357-026-02467-6

**Published:** 2026-08-10

**Authors:** Ákos Bérczi, Fanni É. Szablics, Dóra Hámori, Edit Dósa, Gergő Merkely, Mark Little, Zoltán Ungvári, György Nagy

**Affiliations:** 1 Department of Interventional Radiology, Heart and Vascular Center, Semmelweis University, Városmajor Street 68, Budapest, 1122 Hungary; 2 Hungarian Vascular Radiology Research Group, Heart and Vascular Center, Semmelweis University, Városmajor Street 68, Budapest, 1122 Hungary; 3 Department of Orthopaedic Surgery, Massachusetts General Hospital, Harvard Medical School, 55 Fruit Street, Boston, MA 02114 USA; 4 Department of Radiology, Royal Berkshire NHS Foundation Trust, Royal Berkshire Hospital, Levels 1 and 2, Centre Block, Craven Road, Reading, , Berkshire, RG1 5AN UK; 5 Department of Neurosurgery, Vascular Cognitive Impairment, Neurodegeneration and Healthy Brain Aging Program, University of Oklahoma Health Sciences Center, BRC 1315, 975 NE 10Th Street, Oklahoma City, OK 73104 USA; 6 Department of Rheumatology and Immunology, Semmelweis University, Frankel Leó Street 25–29, Budapest, 1023 Hungary; 7 Heart and Vascular Center, Semmelweis University, Városmajor Street 68, Budapest, 1122 Hungary; 8 Department of Genetics, Cell and Immunobiology, Semmelweis University, Nagyvárad Square 4, Budapest, 1089 Hungary

**Keywords:** Knee osteoarthritis, Geroscience, Joint aging, Healthspan, Cellular senescence, Genicular artery embolization

## Abstract

Knee osteoarthritis (KOA) is a leading cause of late-life pain, mobility loss, and disability, and is increasingly recognized as a phenotype-dependent manifestation of joint aging rather than a purely mechanical disorder. Cellular senescence, inflammaging, mitochondrial dysfunction, synovial inflammation, vascular remodeling, and impaired repair capacity converge to shape KOA symptoms and progression. This narrative review synthesizes evidence on conservative and minimally invasive KOA therapies within a geroscience-guided, phenotype-informed framework. A structured search of PubMed/MEDLINE and Embase from January 2010 to May 2026 identified randomized trials, meta-analyses, guidelines, position statements, and high-quality observational studies evaluating lifestyle interventions, pharmacologic and nutritional strategies, intra-articular therapies, genicular nerve ablation, and genicular artery embolization (GAE). Weight reduction, exercise, and education remain core joint healthspan interventions, with consistent evidence for pain reduction, functional benefit, and favorable systemic effects on inflammation and metabolism. Pharmacologic analgesia, intra-articular corticosteroids, and hyaluronic acid are best interpreted as symptom modulators, while platelet-rich plasma, autologous conditioned serum, and mesenchymal stromal/stem cell-based approaches remain limited by heterogeneity and insufficient long-term structural data. Genicular nerve ablation may support functional longevity through pain control without structural modification. GAE is biologically plausible for synovitis/hypervascularity-dominant KOA, but sham-controlled evidence remains inconsistent. Future trials should distinguish symptomatic relief from true modification of joint aging biology.

**Graphical abstract:**

**Figure:**
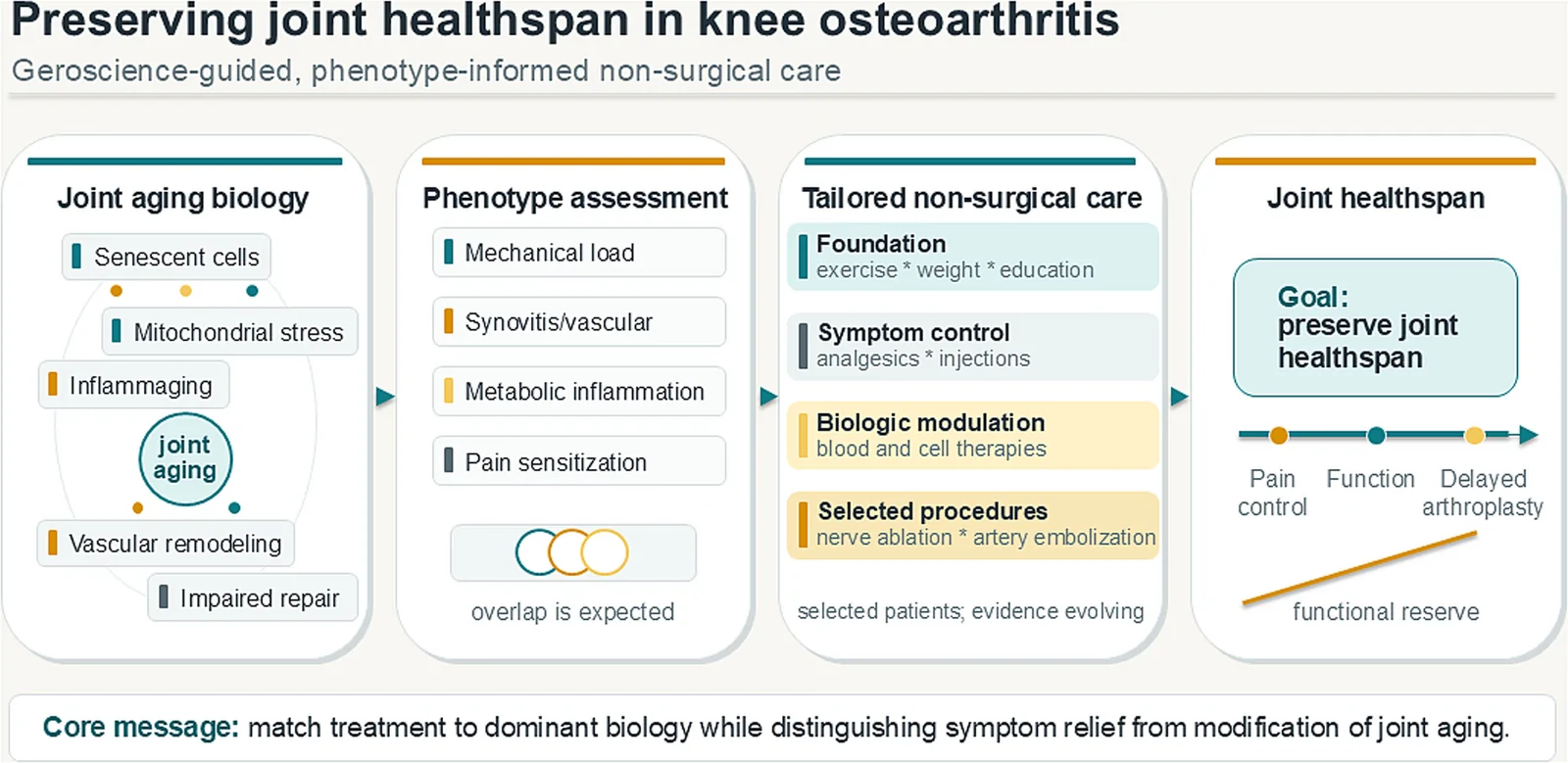

## Introduction

Knee osteoarthritis (KOA), a common clinical phenotype of osteoarthritis (OA), represents a prototypical age-related degenerative disorder and remains one of the leading causes of chronic pain and disability worldwide [1]. As global life expectancy increases, KOA management is increasingly viewed through the lens of geroscience, an interdisciplinary field focused on targeting biological mechanisms of aging to delay, prevent, or mitigate age-associated diseases [2]. Within this framework, KOA is not merely a localized mechanical disorder but also a manifestation of joint senescence driven by inflammaging, cellular dysfunction, mitochondrial stress, vascular changes, and impaired tissue repair capacity.

Conventional treatment paradigms for KOA have historically followed a stepwise escalation from conservative therapy to total joint arthroplasty. This approach is reflected in international guidelines from the American College of Rheumatology/Arthritis Foundation (ACR/AF), the American Academy of Orthopaedic Surgeons (AAOS), the Osteoarthritis Research Society International (OARSI), the European Society for Clinical and Economic Aspects of Osteoporosis, Osteoarthritis and Musculoskeletal Diseases (ESCEO), and the National Institute for Health and Care Excellence (NICE), which position non-surgical management as first-line treatment and reserve surgery for advanced or refractory disease [3–7]. However, a substantial proportion of older adults are either poor surgical candidates because of comorbidities or prefer to delay arthroplasty, highlighting the need to refine minimally invasive and non-surgical strategies that not only alleviate symptoms and restore function but also address age-related molecular, inflammatory, and vascular mechanisms contributing to disease progression [8, 9].

Despite broad agreement on the central role of conservative management, the strength and direction of guideline recommendations vary considerably. Exercise, education, and weight management receive strong support across societies, whereas intra-articular (IA) corticosteroids are typically used for short-term flare control, hyaluronic acid (HA) remains inconsistently supported or discouraged, and orthobiologic therapies such as platelet-rich plasma (PRP) remain controversial because of heterogeneity in trial design, product preparation, and outcome reporting [3–7]. Emerging interventional approaches, including genicular artery embolization (GAE), remain outside major rheumatology and orthopedic OA management guidelines. NICE has issued procedure-specific HealthTech guidance for GAE, concluding that short-term safety raises no major concerns but that evidence on efficacy and long-term safety remains inadequate and that the procedure should be used only in a research context, preferably in randomized controlled trials against sham and current best practice [10]. In contrast, a 2026 Society of Interventional Radiology (SIR) position statement supports GAE as a minimally invasive option for appropriately selected symptomatic KOA patients who have failed conservative therapy and are not candidates for, or wish to delay, total knee arthroplasty, while also emphasizing the need for larger randomized trials [11]. This divergence reflects both evolving evidence and a broader conceptual uncertainty: should KOA treatment remain purely symptom oriented, or should it aim to biologically modify joint aging processes?

**Fig. 1:**
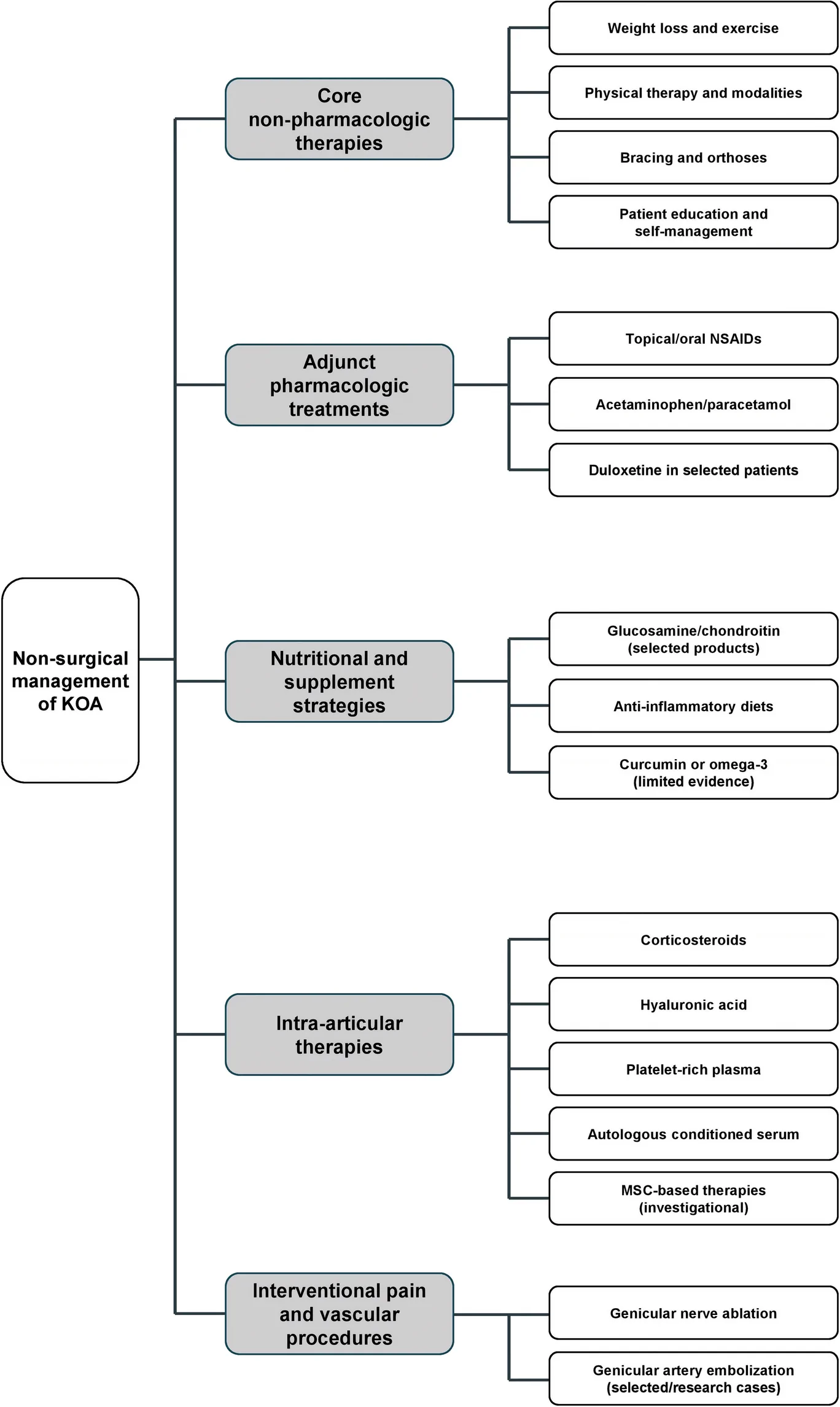
Overview of conservative and minimally invasive non-surgical treatment options for knee osteoarthritis. Genicular artery embolization is shown as a selected or research-context option rather than established standard care. *KOA* knee osteoarthritis, *MSC* mesenchymal stromal/stem cell, *NSAIDs* non-steroidal anti-inflammatory drugs

In this narrative review, we examine the spectrum of conservative and minimally invasive therapies, including metabolic weight optimization, therapeutic exercise, pharmacologic symptom control, IA biologics, genicular nerve ablation (GNA), and emerging vascular interventions (Fig. 1). GAE is discussed not as an established disease-modifying therapy, but as a biologically plausible example of a vascular-targeted strategy for synovitis/hypervascularity-dominant KOA.

Non-surgical KOA management may be reconceptualized as an effort to extend joint healthspan, aligning clinical orthopedics and interventional strategies with the broader objectives of longevity medicine. Joint healthspan can be operationally defined as the duration over which a joint maintains acceptable pain control, functional capacity, and structural integrity without the need for arthroplasty. Unlike traditional OA outcomes, which usually measure pain, function, radiographic progression, or time to arthroplasty at discrete follow-up points, joint healthspan is a longitudinal, integrative construct that combines symptomatic, functional, structural, and biological domains. No universally accepted consensus definition currently exists; therefore, the operational definition used here is intended as a pragmatic framework for research and clinical translation rather than a formal outcome standard. It may be quantified using validated clinical endpoints, including the Western Ontario and McMaster Universities Osteoarthritis Index (WOMAC), Knee Injury and Osteoarthritis Outcome Score (KOOS), visual analog scale (VAS) pain scores, and gait performance; structural imaging markers, including cartilage thickness, bone marrow lesions, and synovitis on magnetic resonance imaging (MRI); and, where available, molecular or inflammatory biomarkers reflecting joint homeostasis. Accordingly, we propose a biologically coherent, phenotype-informed, multimodal treatment framework aimed at optimizing functional outcomes while addressing mechanisms of age-related joint decline rather than merely postponing arthroplasty.

## Methods

A structured narrative literature search was conducted in PubMed/MEDLINE and Embase to identify relevant studies on the non-surgical management of KOA. The search covered publications from January 2010 to May 2026, with additional landmark studies included where appropriate. Search terms included combinations of “knee osteoarthritis,” “KOA,” “non-surgical treatment,” “conservative management,” “intra-articular injection,” “platelet-rich plasma,” “mesenchymal stromal cell,” “mesenchymal stem cell,” “genicular artery embolization,” “radiofrequency ablation,” “genicular nerve ablation,” and “geroscience.” Therapy- and mechanism-specific searches were also performed using terms such as “aging,” “joint aging,” “cellular senescence,” “inflammaging,” “mitochondrial dysfunction,” “synovitis,” “angiogenesis,” “neoangiogenesis,” “weight loss,” “exercise,” “physical therapy,” “bracing,” “duloxetine,” “hyaluronic acid,” “glucosamine,” “chondroitin,” “curcumin,” and “omega-3.” Evidence was synthesized from randomized controlled trials, clinical guidelines, society position statements, systematic reviews, meta-analyses, and selected high-quality observational studies in adult patients with KOA, with emphasis on the mechanistic relevance of established and emerging minimally invasive therapies within a geroscience framework. Non-English publications, case reports, conference abstracts without full text, studies focusing exclusively on surgical interventions, and studies involving non-KOA populations were excluded. Reference lists of selected articles were also screened to identify additional relevant literature. Because this was a narrative rather than systematic review, formal risk-of-bias grading and meta-analysis were not performed.

## Joint aging as a therapeutic target in knee osteoarthritis

OA involves active biological processes, including low-grade inflammation and cellular senescence, that intensify with aging [8, 9, 12]. Senescent cells accumulate in cartilage and synovium and secrete pro-inflammatory and matrix-degrading factors collectively termed the senescence-associated secretory phenotype (SASP), contributing to tissue degeneration [8, 9, 13]. Key age-related signaling pathways, including nuclear factor kappa B, phosphoinositide 3-kinase/protein kinase B/mechanistic target of rapamycin, and Wnt/β-catenin, have been implicated in OA progression [12, 14, 15]. From a geroscience perspective, targeting these mechanisms may offer disease-modifying potential. Senolytic therapies have shown promise in preclinical models, although early clinical trials, including UBX0101, have not demonstrated significant symptomatic benefit [13, 16]. Similarly, systemic geroprotective strategies targeting inflammaging are under investigation, although human evidence remains limited [17, 18]. Emerging systemic geroscience interventions, including senolytics, rapalogs, metformin, nicotinamide adenine dinucleotide boosters, and other geroprotective approaches, may eventually complement local therapies directed at the joint, but they should currently be regarded as investigational for KOA until human phenotype-stratified efficacy and safety data are available [16–18]. Impaired activation of pro-resolving pathways may further contribute to persistent inflammation and failed tissue repair [19].

Within this framework, OA can be conceptualized as the cumulative manifestation of key hallmarks of aging acting on joint tissues. Among these, cellular senescence, inflammaging, mitochondrial dysfunction, and stem cell exhaustion are particularly relevant and provide a biologically structured basis for therapeutic targeting. Cellular senescence drives cartilage degeneration and synovial inflammation through SASP-mediated pathways [8, 9, 13], whereas inflammaging sustains chronic synovitis, cytokine production, and pathological neoangiogenesis [8, 19]. Mitochondrial dysfunction contributes to impaired chondrocyte metabolism and oxidative stress, and stem cell exhaustion limits the regenerative capacity of joint tissues [8, 9].

These hallmarks provide a mechanistic rationale for current and emerging therapies. Exercise and weight reduction attenuate systemic inflammation and improve mitochondrial function [20, 21], whereas IA corticosteroids provide short-term suppression of synovitis [22, 23]. Autologous conditioned serum (ACS) and PRP may modulate cytokine, inflammatory, and anabolic pathways within the senescent microenvironment [24, 25], and mesenchymal stromal/stem cell (MSC)-based therapies aim to restore regenerative signaling, although they remain investigational because of product heterogeneity and limited long-term data [26, 27]. In parallel, a phenotype-informed approach is supported by evidence on KOA pathogenesis [28], interventions targeting nociceptive pathways such as genicular nerve radiofrequency ablation (RFA) [29], trial design recommendations that distinguish symptomatic and structural endpoints [30], structural outcome data from stem cell studies [27], and preclinical evidence supporting the mechanistic plausibility of GAE [31].

Translation of these biological mechanisms into clinical practice requires recognition of OA as a heterogeneous condition comprising distinct yet overlapping phenotypes. Within a geroscience framework, KOA may be broadly categorized into mechanical-dominant, synovitis-dominant, metabolic/inflammaging-dominant, and pain sensitization-dominant phenotypes. Validated clinical criteria for assigning individual patients to these phenotypes remain incompletely standardized; consequently, phenotype labels should be interpreted as pragmatic enrichment constructs rather than rigid diagnostic categories. Mechanical-dominant KOA is driven primarily by abnormal joint loading and biomechanical stress, favoring weight reduction, exercise, and bracing [20, 32]. Metabolic/inflammaging-dominant KOA may coexist with obesity and low-grade systemic inflammation, and nutritional or supplement strategies have been explored with mixed evidence [33, 34]. Synovitis-dominant KOA is characterized by active inflammation and hypervascularity, in which corticosteroids, selected IA therapies, and vascular-targeted interventions such as GAE may be most relevant [22, 31, 35]. Pain sensitization-dominant KOA is driven by altered nociceptive processing and may respond to neuromodulatory therapies or interventional approaches such as GNA [29, 36, 37].

Although these phenotypes frequently overlap within individual patients, this framework enables a shift from symptom-based management toward mechanism-based, phenotype-informed therapeutic strategies. By integrating hallmarks of aging with clinical phenotyping, OA management can be aligned with the principles of precision geroscience, with the overarching aim of preserving joint healthspan and delaying clinically meaningful disease progression. This framework also clarifies a key translational distinction for geroscience: symptomatic benefit, functional healthspan preservation, and structural or molecular modification of joint aging should be evaluated as related but non-equivalent outcomes.

## Conservative non-pharmacologic therapies

Conservative management remains the foundation of KOA care and is particularly important in older adults, in whom maintaining mobility and function while minimizing procedural risk is a central goal. Key approaches include lifestyle modification, physical therapies, bracing, and education. In frail or multimorbid patients, treatment intensity, analgesic exposure, and procedural thresholds should be individualized to preserve mobility while minimizing falls, polypharmacy, renal/cardiovascular risk, and rehabilitation burden.

### Weight loss and exercise

Weight reduction and structured exercise are the most consistently endorsed interventions across major international guidelines. Guidelines and narrative reviews consistently position exercise, weight management, and patient education as first-line management for KOA [3–7, 20].

Randomized controlled trials and evidence syntheses indicate that 5–10% body weight reduction can improve pain and physical function in KOA. Combined diet-induced weight loss and structured exercise produce greater improvements in pain and walking performance than either intervention alone [20, 38]. Exercise programs, including aerobic, strengthening, and flexibility components, also enhance periarticular muscle support and overall functional stability. Current evidence does not establish a single superior exercise modality for older adults with KOA; rather, land-based aerobic activity, strengthening, neuromuscular/balance training, and aquatic exercise can all be appropriate when individualized to pain severity, frailty, balance, cardiometabolic comorbidity, and patient preference [3–7, 20, 21].

From a geroscience perspective, exercise extends beyond symptom control. It reduces systemic inflammaging, improves mitochondrial function, enhances metabolic resilience, and mitigates sarcopenia, mechanisms relevant to the biological context of OA [21]. However, high-quality evidence demonstrating structural disease modification of OA, such as cartilage preservation or reduced joint space narrowing on imaging, remains limited and inconsistent. Exercise should therefore be regarded as a systemic intervention with potential disease-modifying effects at the organismal level rather than a proven structure-modifying therapy for the osteoarthritic joint.

### Physical therapy and modalities

Supervised physical therapy can individualize exercise, improve joint range of motion, and employ adjunctive modalities for pain relief. Common modalities include thermotherapy, transcutaneous electrical nerve stimulation (TENS), ultrasound therapy, neuromuscular electrical stimulation, and balneotherapy. Overall, evidence for these modalities is heterogeneous and generally of low to moderate certainty, with typically small and mainly short-term benefits. TENS, thermotherapy, and balneotherapy show modest symptomatic benefit in some analyses, whereas ultrasound and low-level laser therapy demonstrate inconsistent and limited clinical effects [39–41]. These interventions are best regarded as adjuncts to exercise-based therapy, which remains the core evidence-based conservative treatment for KOA.

### Bracing and orthoses

Offloading knee braces or shoe insoles can redistribute joint forces and reduce pain in KOA, particularly in unicompartmental disease [32, 42]. A valgus unloader brace for medial compartment KOA can modestly improve pain and function, while assistive devices, including canes and walkers, can offload joints and improve stability in frail older patients [32].

### Patient education and self-management

Educating patients about OA, joint protection strategies, and activity pacing is essential. Self-management programs focused on exercise, weight management, and coping strategies can improve outcomes [43]. These strategies empower patients to take an active role in long-term disease management and align with geroscience principles by promoting healthier aging despite arthritis.

## Pharmacologic symptom modulation

Although beyond the primary scope of this review, pharmacologic analgesia warrants brief mention because symptom control can enable continued physical activity. Topical and oral non-steroidal anti-inflammatory drugs (NSAIDs), such as diclofenac gel, can reduce OA pain and remain recommended first-line pharmacologic therapies for many patients [3, 6, 44]. However, chronic NSAID therapy in older adults is constrained by increased risks of gastrointestinal bleeding, renal impairment, and adverse cardiovascular events [44]. Acetaminophen/paracetamol is safer for some patients but often insufficient for moderate-to-severe pain. Duloxetine, a serotonin–norepinephrine reuptake inhibitor, has shown modest efficacy for chronic OA pain and may be considered, particularly when centralized pain is suspected [6, 36]. Opioids are generally avoided in chronic OA because of adverse effects and lack of long-term benefit; in a randomized trial, opioid therapy was not superior to non-opioid medication therapy for pain-related function in chronic back pain, hip OA pain, or knee OA pain [45]. In a geroscience context, these drugs treat symptoms but do not alter the course of OA; they should therefore be viewed as adjuncts that enable patients to remain active in exercise therapy rather than as standalone long-term solutions.

## Nutritional and supplement interventions

Numerous nutritional supplements and dietary strategies have been explored in OA with the goal of modulating inflammation and supporting cartilage health.

### Glucosamine and chondroitin

Glucosamine and chondroitin have been extensively studied, with mixed findings. Early evidence suggested modest symptomatic or structural benefits, whereas many later studies reported no meaningful effect beyond placebo [46]. Owing to this inconsistency, the 2019 ACR/AF guideline strongly recommends against glucosamine and conditionally recommends against chondroitin in KOA [6]. In contrast, the MOVES trial reported that combined glucosamine and chondroitin was as effective as celecoxib for KOA pain over 6 months, and ESCEO supports selected pharmaceutical-grade preparations [5, 33]. Thus, routine use is not supported by most North American guidelines, whereas ESCEO remains more favorable to selected pharmaceutical-grade products.

### Anti-inflammatory diets and selected supplements

Anti-inflammatory dietary approaches, including Mediterranean-style diets rich in fruits, vegetables, whole grains, and omega-3-containing fish, are sometimes adopted by patients with OA [34]. Although these diets provide clear cardiometabolic benefits, evidence for direct symptomatic improvement in OA is limited and is primarily derived from observational studies and small interventional trials. Their potential benefit is likely indirect, mediated through weight reduction and attenuation of low-grade systemic inflammation. Nutritional supplements, including curcumin and omega-3 fatty acids, have shown small short-term improvements in pain and function in randomized trials, but the certainty of evidence is low because of heterogeneity in formulations, dosing, and study design [47]. Robust evidence from large, long-term randomized trials demonstrating sustained or structure-modifying effects is lacking. Within a geroscience framework, nutrition primarily addresses systemic metabolic and inflammatory drivers of joint aging rather than directly modifying joint structure.

## Intra-articular and biologic injection strategies

IA administration of therapeutic agents represents a cornerstone of minimally invasive treatment strategies for OA. Such injections allow localized symptom control with limited systemic exposure. Corticosteroids and HA remain the most commonly used IA therapies, whereas biologic approaches, including PRP and cell-based injections, are increasingly explored in clinical practice.

### Corticosteroid injections

IA corticosteroids, such as triamcinolone, have long been used to reduce inflammation and pain in OA. They often yield prompt pain relief, peaking approximately 1–4 weeks after injection, but benefits are usually temporary and commonly diminish within weeks to a few months [22, 23]. Repeated steroid injections may diminish in effect and may carry risks, including accelerated cartilage loss, subchondral bone changes, and systemic adverse effects such as hyperglycemia in patients with diabetes if administered too frequently. Although specific limits vary across practices, repeated injections should be used cautiously and individualized according to disease severity, comorbidity, and alternative treatment options [6]. Despite short-term efficacy, steroids do not modify disease progression and frequent use may be unfavorable to cartilage; thus, they are best reserved for flare management or for bridging patients to longer-term strategies.

### Hyaluronic acid injections

IA HA, or viscosupplementation, involves injection of synthetic or biologically derived HA to restore the viscoelastic properties of synovial fluid and potentially exert chondroprotective effects. Treatment protocols typically consist of 3–5 weekly injections or a single high-molecular-weight preparation. Meta-analyses of randomized trials demonstrate small improvements in pain and function compared with placebo, with effects that often approach or fall below minimal clinically important difference thresholds [48]. Comparative analyses suggest that corticosteroids may provide greater short-term pain relief, whereas HA may provide more durable improvement up to approximately 6 months in some studies [49]. Guideline recommendations therefore diverge: AAOS does not recommend routine IA HA use, ACR/AF conditionally recommends against IA HA for KOA, NICE recommends against IA hyaluronan injections, OARSI conditionally recommends use in selected patients depending on comorbidity status, and ESCEO supports selected pharmaceutical-grade preparations [3–7]. HA injections are generally safe, with transient post-injection flares as the most common adverse event. Given the modest effect sizes and variability in response, HA should be considered selectively, primarily in patients with early-to-moderate disease who have not achieved sufficient improvement with first-line conservative therapy, after transparent discussion that expected benefits are typically small.

### Platelet-rich plasma injections

PRP occupies an intermediate zone between purely symptomatic therapy and biologically active intervention. As an autologous blood-derived product enriched in platelets and growth factors, PRP is injected intra-articularly to promote tissue repair and modulate inflammation [24]. Biologically, PRP releases mediators such as transforming growth factor β, platelet-derived growth factor, and insulin-like growth factor 1, which may influence cartilage homeostasis and inflammatory signaling. Clinical evidence has expanded substantially over the past decade, with multiple randomized controlled trials and meta-analyses demonstrating improvements in pain and function compared with placebo, with effects observed up to 12 months in some studies [24]. However, the magnitude of benefit is variable, and risk of bias and substantial heterogeneity, including differences in platelet concentration, leukocyte content, preparation methods, injection protocols, and comparator groups, limit certainty and generalizability. Notably, the high-quality blinded RESTORE trial did not demonstrate superiority of PRP over saline placebo for pain or medial tibial cartilage volume [50]. Exploratory subgroup analyses suggest that higher platelet concentrations may be associated with greater or more sustained symptom improvement; however, these findings are hypothesis generating and should not be interpreted as causal given the lack of standardization and direct comparative trials [24]. Reflecting these uncertainties, guideline bodies remain cautious: AAOS reports limited evidence, ACR/AF strongly recommends against PRP in knee and hip OA, and OARSI does not formally endorse PRP [3, 4, 6]. Within a geroscience framework, PRP may be viewed as a biologically plausible intervention targeting inflammatory and catabolic pathways, but its clinical role remains constrained by methodological variability and incomplete standardization.

### Autologous conditioned serum

ACS, often known by the trade name Orthokine, involves incubating a patient’s blood to induce high concentrations of interleukin-1 (IL-1) receptor antagonist and growth factors, followed by IA injection of the serum [51]. The rationale is to counteract IL-1, a key inflammatory cytokine in OA. A randomized trial-based meta-analysis by Curtis et al. suggested that ACS may reduce pain compared with pooled comparators; however, when compared specifically with saline, differences in WOMAC and VAS scores at 6 months were not significant [25, 51]. Overall, evidence remains promising but insufficient; large-scale data are limited, and preparation is specialized. ACS is not yet widely adopted, but it exemplifies the broader trend of using autologous biologics to modify the IA environment.

### Mesenchymal stromal/stem cell-based therapies

MSC-based therapy for OA typically involves harvesting MSCs from bone marrow or adipose tissue and administering them intra-articularly [26]. These therapies are hypothesized to exert predominantly paracrine immunomodulatory and anti-inflammatory effects rather than direct cartilage regeneration. Clinical evidence from randomized trials and meta-analyses suggests improvements in pain, function, and quality of life compared with controls, with benefits observed up to 6–12 months in some studies [26]. However, effect sizes vary, and heterogeneity across studies remains substantial. MSC-based therapies encompass a broad spectrum of products, including culture-expanded cells and minimally manipulated preparations such as stromal vascular fraction, with significant variability in cell source, processing methods, dosing, and injection protocols. Subgroup analyses suggesting differential effects by dose or tissue source remain exploratory and are not based on definitive direct comparative trials. Many MSC-based therapies remain investigational, and regulatory status varies considerably across countries. Reported adverse events are generally mild and transient, such as pain or swelling at the injection site, but long-term safety data remain limited. Theoretical and incompletely characterized risks include infection, ectopic tissue formation, and tumorigenicity, although these have not been consistently observed in clinical studies to date [27]. Some studies have reported improvements in cartilage quality on MRI or delayed joint space narrowing; however, robust evidence for structural disease modification is lacking [27]. Consequently, major guidelines have not endorsed MSC-based therapies and often classify them as experimental, with ACR/AF strongly recommending against stem cell injections in knee and hip OA because of heterogeneity, lack of standardization, and insufficient evidence [6]. The conceptual appeal of MSC-based therapies lies in their alignment with stem cell exhaustion as a hallmark of aging, but clinical translation remains limited by regulatory, methodological, and biological uncertainties.

## Genicular nerve ablation

GNA is a minimally invasive intervention for patients with persistent KOA pain despite conservative therapy. The procedure targets sensory articular branches, typically the superior medial, superior lateral, inferior medial, and in many protocols the inferior lateral genicular nerves, using radiofrequency energy to disrupt nociceptive transmission while preserving motor function [29, 37]. In most clinical protocols, diagnostic genicular nerve blocks are performed before RFA to identify suitable responders. A positive block is generally defined as at least 50% temporary pain relief, often within 24–48 h, although some centers apply stricter thresholds of 70–80% to optimize patient selection. The procedure is performed under imaging guidance, usually fluoroscopy or ultrasound, typically in an outpatient setting, with pain relief emerging over several weeks and lasting approximately 6–12 months, consistent with nerve regeneration timelines.

Randomized trials have demonstrated superiority over sham procedures in short- to mid-term follow-up. A systematic review including more than 2200 patients reported that approximately 50% achieved at least 50% pain reduction at 6 months, with sustained benefit in a substantial subset at 12 months; larger-lesion techniques, such as cooled RFA, may be associated with improved durability [37]. Functional improvements, including better WOMAC scores and walking capacity, are also reported [29]. The procedure has a favorable safety profile, with mainly minor and transient local adverse effects. Guideline bodies remain cautious but not uniformly negative: ACR/AF conditionally recommends RFA for KOA, whereas AAOS classifies denervation therapy as a limited-evidence option that may reduce pain and improve function, and OARSI does not include RFA as a core recommended treatment [3, 4, 6]. Although RFA does not modify joint structure, it occupies an important conceptual role. From a geroscience perspective, effective pain control may support functional longevity by enabling sustained physical activity, thereby indirectly contributing to muscle preservation, metabolic health, and reduction of systemic inflammaging. Thus, while not disease-modifying in a structural sense, GNA may contribute to preservation of functional healthspan in carefully selected patients who are not candidates for, or wish to delay, arthroplasty.

## Genicular artery embolization

GAE is an emerging, image-guided endovascular therapy for symptomatic KOA that targets the vascular–inflammatory component of the disease and is therefore conceptually aligned with a geroscience framework. First described in Japan in 2014, GAE has gained international interest as a non-surgical option for patients with persistent pain and synovitis who wish to delay arthroplasty or are poor surgical candidates [31, 52–54].

### Rationale and mechanism

OA is increasingly recognized as a biologically active disorder characterized by synovial inflammation and neoangiogenesis, with hypervascular synovial networks amplifying inflammatory mediator production and facilitating nociceptive nerve ingrowth [28, 35, 55]. Angiographic blush correlates with regions of synovitis identified on MRI and Doppler ultrasound, and several studies have demonstrated an association between synovial hypervascularity and pain severity, supporting the biological rationale for vascular-targeted therapy [35, 55]. GAE aims to selectively embolize these abnormal neovessels, thereby reducing pathologic perfusion and downregulating local inflammatory signaling.

### Patient selection

Current evidence supports an emerging target population. GAE is most commonly performed in patients with Kellgren–Lawrence grade 1–3 KOA, persistent moderate-to-severe pain, for example a VAS score of at least 5, despite optimized conservative therapy, including exercise, analgesics, and often prior IA injections, and imaging evidence of active synovitis [31, 52–56]. Synovitis-dominant phenotypes are typically operationalized using contrast-enhanced MRI, including synovial thickening and enhancement scores, or Doppler ultrasound, including hypervascular signal grades, although thresholds are not fully standardized. Greater synovitis has been associated with improved response in observational studies; however, predictive performance has not been prospectively validated, and selection criteria remain partly empirical [52, 55, 56].

Candidate selection must also consider features that reduce expected benefit or increase procedural risk. Patients with end-stage mechanically driven deformity, advanced Kellgren–Lawrence grade 4 disease without active synovitis, non-OA pain generators, or pain sensitization-dominant presentations may be less likely to benefit from a vascular-targeted intervention. Relative or absolute contraindications include active infection, severe peripheral arterial disease or compromised cutaneous collateral supply, uncorrected coagulopathy, severe renal dysfunction, and high-risk iodinated contrast allergy. These considerations reinforce the need for multidisciplinary assessment and imaging-based confirmation of a synovitis/hypervascularity-dominant phenotype before GAE [52, 53, 55].

### Procedure and technical aspects

GAE is typically performed under local anesthesia with optional conscious sedation via femoral arterial access. Selective angiography of genicular branches from the superficial femoral and/or popliteal artery is used to identify pathologic hypervascular blush, followed by superselective microcatheterization [53–55]. Embolic materials across studies include calibrated microspheres or particles, most commonly in the 100–300 µm or 300–500 µm range, and less commonly imipenem/cilastatin particles or other temporary embolic agents. Embolization is performed slowly to reduce abnormal blush while avoiding non-target embolization. Periprocedural management typically includes local anesthesia, mild sedation, and post-procedural oral analgesia, including NSAIDs with or without short-course opioids; protocols vary across centers [52–56].

### Effectiveness

Clinical outcomes are derived primarily from early single-arm cohorts and a limited number of controlled studies. A systematic review and meta-analysis by Taslakian et al. including nine studies and 339 knees reported improvements from baseline to follow-up at 12 months, including a mean VAS reduction of approximately 36 points on a 0–100 scale and WOMAC improvement of approximately 34 points [55]. These changes exceed commonly accepted minimal clinically important difference thresholds. However, most included studies were non-randomized, limiting causal inference and increasing susceptibility to placebo effects and selection bias. More recent evidence syntheses, including a systematic review of three sham-controlled randomized trials including 138 patients by Milhem et al., provide a more methodologically robust assessment [57]. The randomized trials differed in patient enrichment for synovitis/hypervascularity, baseline OA severity, use of contrast-enhanced MRI or angiographic blush as eligibility criteria, embolic material and particle size, sham technique, and primary outcome timing, which likely contributed to discordant results. Short-term significant pain reduction at 4 months was observed in one of the three included trials, whereas the remaining studies demonstrated no statistically significant differences between GAE and sham procedures in KOOS or VAS outcomes at mid-term follow-up ranging from 4 to 12 months. This uncertainty was reinforced by the 12-month sham-controlled trial reported by van Zadelhoff et al., in which 58 patients with mild-to-moderate KOA showed sustained pain improvement in both GAE and sham groups, without significant between-group differences in KOOS pain, VAS pain, quality of life, or MRI-assessed synovitis [58]. Thus, although open-label and single-arm studies continue to report clinically meaningful symptomatic improvements suggesting a possible therapeutic signal, sham-comparator trials do not yet establish reproducible superiority over placebo or other non-specific procedural effects. Interpretation of efficacy should therefore explicitly account for substantial uncertainty regarding the magnitude of benefit attributable to embolization itself.

### Safety, radiation, and contrast exposure

Technical success rates approach 99–100% in published series [55]. Adverse events are predominantly minor, including transient pain flare, skin discoloration or erythema, and access site complications. Rare but clinically relevant complications include skin ulceration or necrosis, transient nerve injury, and very rarely osteonecrosis, typically related to non-target embolization. Procedure-related exposure is modest: reported radiation doses are generally in the range of approximately 5–20 Gy·cm^2^ as dose-area product, and contrast volumes are approximately 50–100 mL, comparable to other peripheral embolization procedures. These parameters vary depending on operator experience and procedural complexity [52–55].

### Evidence position and geroscience interpretation

Available data suggest greater responsiveness in synovitis-dominant OA, whereas end-stage, mechanically driven disease, particularly Kellgren–Lawrence grade 4 disease with minimal synovitis, appears less responsive. However, formal predictive models integrating imaging biomarkers, including MRI synovitis scores and Doppler grading, with clinical outcomes are not yet validated, and prospective stratified trials are needed. Broad OA management guidelines from ACR/AF, AAOS, OARSI, and NICE NG226 have not yet incorporated GAE because of limited high-quality randomized evidence and cost-effectiveness data [3, 4, 6, 7, 57, 58]. NICE procedure-specific HealthTech guidance states that short-term safety raises no major concerns but that evidence on efficacy and long-term safety is inadequate in quality and quantity; NICE therefore recommends research-context use, preferably in randomized controlled trials against sham and current best practice [10]. In contrast, the 2026 SIR position statement supports GAE for appropriately selected symptomatic KOA patients after failure of conservative therapy, particularly when arthroplasty is unsuitable or intentionally delayed, while also calling for larger randomized trials [11]. Within a geroscience framework, GAE is best understood not primarily as a technical innovation but as a vascular-targeted intervention addressing the angiogenesis–inflammation axis of joint aging.

**Fig. 2:**
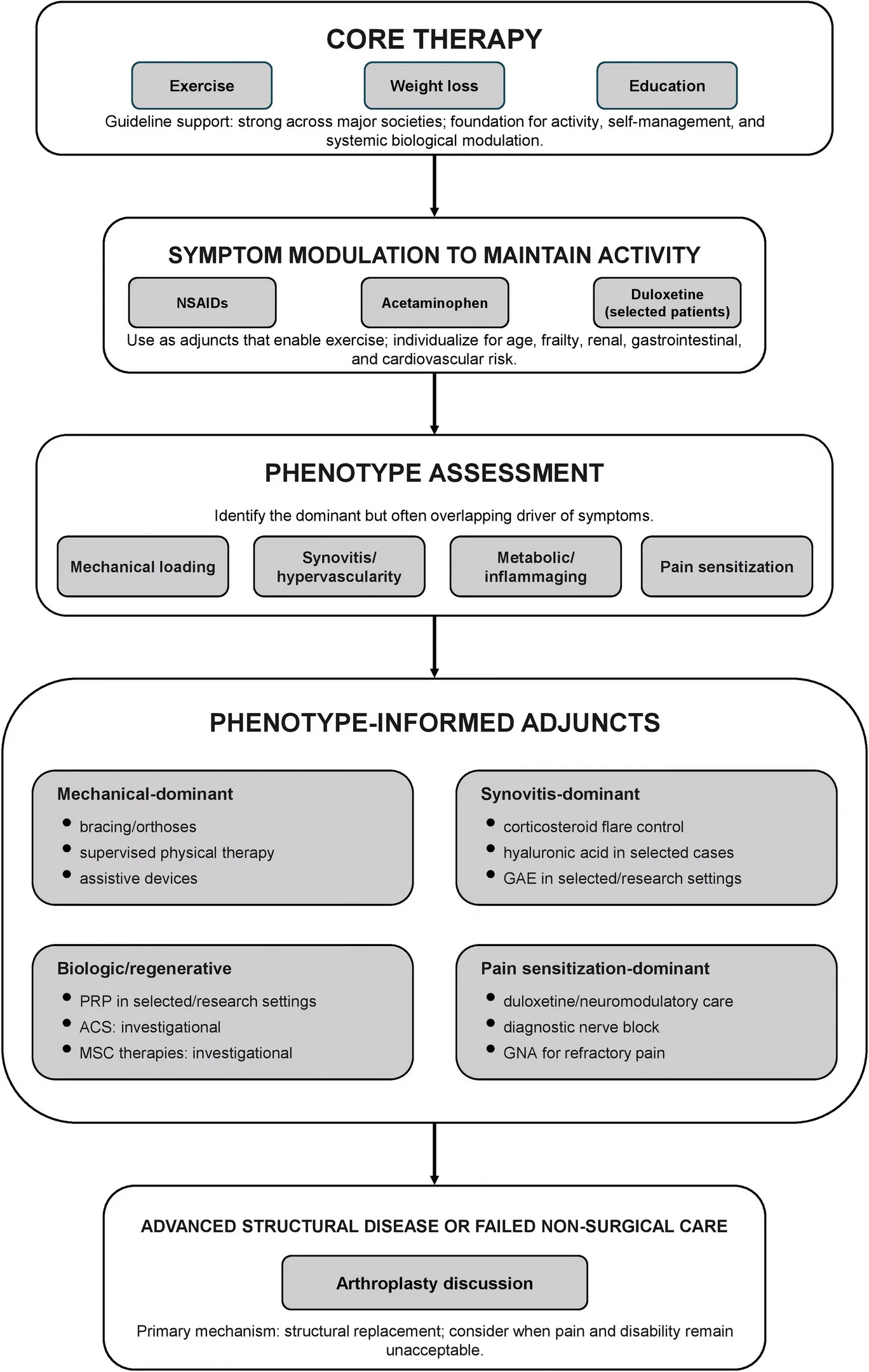
Phenotype-informed algorithm for non-surgical treatment selection in knee osteoarthritis. Genicular artery embolization should be interpreted as a selected or research-context option rather than established standard care. *ACS* autologous conditioned serum, *GAE* genicular artery embolization, *GNA* genicular nerve ablation, *MSC* mesenchymal stromal/stem cell, *NSAIDs* non-steroidal anti-inflammatory drugs, *PRP* platelet-rich plasma

Taken together, these findings support a phenotype-informed, mechanism-based approach to treatment selection, summarized in the proposed management algorithm (Fig. 2).

An integrated summary of the main treatment categories, best-fit phenotypes, current evidence positions, geroscience relevance, and key limitations is provided in Table 1.

**Table 1:** Mechanism- and phenotype-informed summary of non-surgical therapies for knee osteoarthritis

Therapy/category	Best-fit phenotype or clinical context	Current evidence/guideline position	Geroscience relevance	Main limitation
Weight reduction, structured exercise, and physical therapy	KOA associated with mechanical load, metabolic factors, or frailty; foundational care for most patients	Strong guideline support as core therapy across major societies; improves pain, function, and mobility	Reduces joint loading and systemic inflammaging; improves metabolic and functional reserve	Requires sustained adherence; benefits may be limited by frailty, severe pain, or comorbidities
Education, self-management, bracing, and orthoses	Malalignment, activity-related pain, instability, or patients requiring low-risk adjuncts	Generally supported as adjunctive conservative care; magnitude of benefit varies by phenotype and device fit	Supports activity maintenance, fall-risk reduction, and preservation of joint healthspan	Heterogeneous interventions; adherence, comfort, and access influence effectiveness
Topical/oral analgesics, NSAIDs, acetaminophen, and duloxetine	Symptom-dominant KOA, inflammatory flares, or centralized pain features requiring pharmacologic control	Useful for symptom management; topical NSAIDs often favored in older adults; systemic therapy requires risk stratification	Primarily symptom-modifying rather than mechanism- or structure-modifying	Gastrointestinal, renal, cardiovascular, hepatic, and drug-interaction concerns in older adults
IA corticosteroids	Short-term inflammatory flare or effusion-dominant KOA	Short-term pain relief; recommendations vary, and repeated use requires caution	Transient suppression of synovial inflammation but limited evidence for altering joint aging biology	Durability is limited; repeated injections may raise cartilage and systemic safety concerns
HA	Selected mild-to-moderate KOA with lubrication/synovial homeostasis phenotype	Evidence and guideline positions are inconsistent; some guidelines discourage routine use	Potential viscoelastic and anti-inflammatory effects within the synovial microenvironment	Modest average effect size, delayed onset, cost, and heterogeneous preparations
PRP and ACS	Early-to-moderate KOA with inflammatory or reparative phenotype; often considered after failure of core therapy	Promising but heterogeneous evidence; routine use remains debated because of preparation and protocol variability	May modulate cytokine balance, catabolic signaling, and local repair responses	Lack of standardization, variable biologic composition, and insufficient long-term structural endpoints
MSC-based and other cell-derived therapies	Investigational option for selected patients with reparative or inflammatory phenotypes	Biologically plausible, but insufficient standardization and regulatory uncertainty limit routine clinical use	Paracrine immunomodulation and tissue repair signaling are aligned with geroscience concepts	Limited high-quality long-term trials; unclear dose, source, processing, and true disease-modifying effect
GNA/RFA	Refractory nociceptive pain or pain sensitization-dominant phenotype when surgery is unsuitable or delayed	Can improve pain in selected patients; evidence is mainly symptom-focused and protocol-dependent	Preserves function through pain reduction but does not target joint senescence or structural degeneration	No demonstrated structural modification; nerve targets, technique, and durability vary
GAE	Synovitis- or hypervascularity-dominant KOA after failed conservative therapy; arthroplasty unsuitable or intentionally delayed	NICE procedure-specific guidance recommends research-only use; SIR supports selected use after failed conservative therapy; sham-controlled evidence remains inconsistent, including negative 12-month data	Targets the vascular–inflammatory axis of joint aging and synovial hypervascularity	Optimal patient selection, durability, cost-effectiveness, placebo contribution, and long-term safety require further trials

*ACS* autologous conditioned serum, *GAE* genicular artery embolization, *GNA* genicular nerve ablation, *HA* hyaluronic acid, *IA* intra-articular, *KOA* knee osteoarthritis, *MSC* mesenchymal stromal/stem cell, *NICE* National Institute for health and care excellence, *NSAIDs* non-steroidal anti-inflammatory drugs, *PRP* platelet-rich plasma, *RFA* radiofrequency ablation, *SIR* society of interventional radiology

Evidence positions are summarized narratively from the cited sources and do not represent formal Grading of Recommendations Assessment, Development and Evaluation (GRADE) ratings. Because many patients exhibit overlapping phenotypes, a single patient may fit more than one treatment category; the table should therefore be interpreted as a guide to multimodal, phenotype-informed selection rather than as mutually exclusive categories

## Future directions for geroscience-aligned KOA trials

Future studies should shift from treatment-class trials to mechanism- and phenotype-stratified trials. The most immediate priority is to predefine strata based on mechanical load, synovitis/hypervascularity, metabolic/inflammaging, and pain sensitization, using clinical phenotyping plus imaging biomarkers, and to report whether benefit differs by phenotype. Future precision strategies should test circulating inflammatory markers, synovial fluid mediators, synovitis, and cartilage measures derived from MRI or ultrasound, radiomic features, and composite biological aging biomarkers as predictors of treatment response. Artificial intelligence, radiomics, and machine learning models may further support reproducible phenotype classification and individualized patient selection, provided that algorithms are prospectively validated and clinically interpretable. For GAE, sham-controlled designs should enrich for synovitis/hypervascularity and incorporate MRI or Doppler endpoints alongside clinical outcomes.

Second, trials should distinguish analgesia from biological modification of joint aging. A minimum geroscience-aligned endpoint set should combine patient-reported pain and function, objective mobility or gait measures, muscle strength and frailty metrics, structural MRI markers, and inflammatory or senescence-associated biomarkers where feasible. Follow-up should also assess durability, arthroplasty-free survival, and long-term preservation of joint healthspan.

Third, supportive interventions such as exercise, weight loss, bracing, and analgesia should be evaluated as components of joint healthspan programs rather than only as isolated symptom treatments. Combination strategies should be tested explicitly, because lifestyle interventions may improve systemic resilience while PRP, GNA, or GAE address local inflammatory, nociceptive, or vascular targets; such multimodal programs may be especially relevant for older adults with overlapping phenotypes. In this design, symptom modulators are judged by whether they maintain activity, reduce immobility-related frailty, and attenuate systemic inflammaging.

Finally, biologic and vascular interventions require standardized product or procedure descriptions, long-term follow-up, adverse event surveillance, and cost-effectiveness analyses before routine adoption. This is particularly important for interventions that are mechanistically plausible but whose current clinical effects may be strongly influenced by placebo responses or heterogeneous patient selection.

## Limitations

This review has several limitations. First, the literature search and synthesis were narrative rather than systematic; therefore, formal risk-of-bias assessment and quantitative evidence grading were not performed. Second, there is substantial heterogeneity across studies, particularly for biologic therapies such as PRP, ACS, and MSC-based interventions, where variability in product composition, including platelet concentration, leukocyte content, cell source, processing methods, dosing, and administration protocols, limits comparability and generalizability. Third, for several minimally invasive procedures, including GAE and GNA, the availability of high-quality sham-controlled randomized trials remains limited, and recent GAE data reinforce the potential for sustained placebo responses in invasive pain procedures. Fourth, the evidence base is subject to publication bias, with a predominance of positive or early-phase studies and relative underreporting of negative findings. Fifth, many studies focus on patients with mild-to-moderate OA, limiting extrapolation to advanced disease, such as Kellgren–Lawrence grade 4 disease. Additionally, inconsistencies in outcome measures and limited use of validated structural endpoints, including imaging-based progression, constrain assessment of disease-modifying effects. Finally, short- to mid-term follow-up predominates, and long-term safety and durability of emerging therapies remain insufficiently characterized.

## Conclusions

The management of KOA has evolved beyond simple analgesia and inevitable joint replacement. A broad spectrum of conservative and minimally invasive therapies now exists to preserve mobility and quality of life, particularly in older adults. Lifestyle interventions, most notably weight reduction and structured exercise, remain the cornerstone of treatment, supported by strong guideline endorsement and robust evidence for symptom improvement and metabolic benefit [3–6, 20]. Injectable therapies, including IA corticosteroids and HA, provide short-term or modest symptomatic relief, although effect sizes are variable across patient subgroups. Orthobiologic approaches such as PRP, ACS, and MSC-based therapies show biological plausibility and emerging evidence of clinical benefit; however, heterogeneity, limited standardization, regulatory uncertainty, and insufficient long-term data constrain routine use [24–26].

Interventional procedures, including GNA and GAE, have further expanded the therapeutic landscape for refractory KOA. These modalities have shown encouraging short- to mid-term improvements in selected studies, but they do not constitute established disease-modifying standards of care, and evidence for structural modification and long-term comparative effectiveness remains limited [37, 55, 57, 58]. GAE now has specialty society support for carefully selected patients after failed conservative therapy, particularly when arthroplasty is unsuitable or intentionally delayed; however, sham-controlled trials have not consistently demonstrated superiority over placebo procedures, reinforcing the need for larger phenotype-stratified randomized studies [11, 58]. These interventions should therefore be considered promising, selective options for carefully chosen refractory cases, preferably in specialized centers or clinical research settings.

OA care should be reconceptualized not as a linear pathway toward arthroplasty but as a phenotype-informed, mechanism-driven continuum. Integrating mechanical, synovitis-dominant, metabolic/inflammaging-dominant, and pain sensitization domains provides a more biologically coherent framework for treatment selection than traditional stepwise escalation. Within this paradigm, conservative and minimally invasive therapies may contribute to preservation of joint healthspan, defined by sustained pain control, functional capacity, and delayed need for joint replacement.

A multimodal, phenotype-informed strategy is likely to be most effective, combining lifestyle interventions with adjunctive pharmacologic and minimally invasive therapies tailored to underlying disease mechanisms. However, despite conceptual advances, current evidence still supports a predominantly symptomatic treatment paradigm. Realizing a true shift toward disease-modifying, geroscience-aligned care will require phenotype-stratified clinical trials, validated imaging and molecular biomarkers, and clearer distinction between symptomatic, functional, structural, and molecular endpoints. In this context, emerging biologically aligned interventions, including vascular-targeted therapies such as GAE, may help bridge the gap between symptom control and mechanism-based modification of joint aging. Future randomized trials should therefore evaluate not only pain and functional outcomes but also biomarkers of biological joint aging, objective mobility, frailty, structural progression, arthroplasty-free survival, and long-term preservation of joint healthspan.

## Data Availability

No new datasets were generated or analyzed for this narrative review.

## Declarations

### Use of generative artificial intelligence (AI) and AI-assisted technologies

During the preparation of this work, the authors used OpenAI ChatGPT to support language editing.

## References

[1] Cui A, Li H, Wang D, Zhong J, Chen Y, Lu H. Global, regional prevalence, incidence and risk factors of knee osteoarthritis in population-based studies. EClinicalMedicine. 2020;29–30. 10.1016/j.eclinm.2020.100587. PMC7704420 34505846

[2] Kaeberlein M. Translational geroscience: a new paradigm for 21st century medicine. Transl Med Aging. 2017;1:1–4. 10.1016/j.tma.2017.09.004. PMC7098696 32219192

[3] Bannuru RR, Osani MC, Vaysbrot EE, Arden NK, Bennell K, Bierma-Zeinstra SMA, Kraus VB, Lohmander LS, Abbott JH, Bhandari M, Blanco FJ, Espinosa R, Haugen IK, Lin J, Mandl LA, Moilanen E, Nakamura N, Snyder-Mackler L, Trojian T, Underwood M, McAlindon TE. OARSI guidelines for the non-surgical management of knee, hip, and polyarticular osteoarthritis. Osteoarthritis Cartilage. 2019;27:1578–89. 10.1016/j.joca.2019.06.011. 31278997

[4] Brophy RH, Fillingham YA. AAOS clinical practice guideline summary: management of osteoarthritis of the knee (nonarthroplasty), third edition. J Am Acad Orthop Surg. 2022;30:e721–9. 10.5435/JAAOS-D-21-01233. 35383651

[5] Bruyere O, Honvo G, Veronese N, Arden NK, Branco J, Curtis EM, Al-Daghri NM, Herrero-Beaumont G, Martel-Pelletier J, Pelletier JP, Rannou F, Rizzoli R, Roth R, Uebelhart D, Cooper C, Reginster JY. An updated algorithm recommendation for the management of knee osteoarthritis from the European Society for Clinical and Economic Aspects of Osteoporosis, Osteoarthritis and Musculoskeletal Diseases (ESCEO). Semin Arthritis Rheum. 2019;49:337–50. 10.1016/j.semarthrit.2019.04.008. 31126594

[6] Kolasinski SL, Neogi T, Hochberg MC, Oatis C, Guyatt G, Block J, Callahan L, Copenhaver C, Dodge C, Felson D, Gellar K, Harvey WF, Hawker G, Herzig E, Kwoh CK, Nelson AE, Samuels J, Scanzello C, White D, Wise B, Altman RD, DiRenzo D, Fontanarosa J, Giradi G, Ishimori M, Misra D, Shah AA, Shmagel AK, Thoma LM, Turgunbaev M, Turner AS, Reston J. 2019 American College of Rheumatology/Arthritis Foundation guideline for the management of osteoarthritis of the hand, hip, and knee. Arthritis Rheumatol. 2020;72:220–33. 10.1002/art.41142. PMC10518852 31908163

[7] National Institute for Health and Care Excellence, 2022. Osteoarthritis in over 16s: diagnosis and management. NICE Guideline NG226. https://www.nice.org.uk/guidance/ng226 (accessed 15 May 2026). 36745715

[8] Coryell PR, Diekman BO, Loeser RF. Mechanisms and therapeutic implications of cellular senescence in osteoarthritis. Nat Rev Rheumatol. 2021;17:47–57. 10.1038/s41584-020-00533-7. PMC8035495 33208917

[9] Xie J, Wang Y, Lu L, Liu L, Yu X, Pei F. Cellular senescence in knee osteoarthritis: molecular mechanisms and therapeutic implications. Ageing Res Rev. 2021;70. 10.1016/j.arr.2021.101413. 34298194

[10] National Institute for Health and Care Excellence, 2021. Genicular artery embolisation for pain from knee osteoarthritis. NICE HealthTech guidance HTG595 (formerly Interventional Procedures Guidance IPG708). https://www.nice.org.uk/guidance/htg595 (accessed 15 May 2026).

[11] Ahmed O, Taslakian B, Okuno Y, Minko P, Golzarian J, Bagla S, Sapoval M, Correa MP, Little M. Society of interventional radiology position statement on genicular artery embolization for symptomatic knee osteoarthritis. J Vasc Interv Radiol. 2026. 10.1016/j.jvir.2026.108803. 42118083

[12] Rigoglou S, Papavassiliou AG. The NF-kappaB signalling pathway in osteoarthritis. Int J Biochem Cell Biol. 2013;45:2580–4. 10.1016/j.biocel.2013.08.018. 24004831

[13] Jeon OH, Kim C, Laberge RM, Demaria M, Rathod S, Vasserot AP, Chung JW, Kim DH, Poon Y, David N, Baker DJ, van Deursen JM, Campisi J, Elisseeff JH. Local clearance of senescent cells attenuates the development of post-traumatic osteoarthritis and creates a pro-regenerative environment. Nat Med. 2017;23:775–81. 10.1038/nm.4324. PMC5785239 28436958

[14] Sun K, Luo J, Guo J, Yao X, Jing X, Guo F. The PI3K/AKT/mTOR signaling pathway in osteoarthritis: a narrative review. Osteoarthritis Cartilage. 2020;28:400–9. 10.1016/j.joca.2020.02.027. 32081707

[15] Yazici Y, McAlindon TE, Fleischmann R, Gibofsky A, Lane NE, Kivitz AJ, Skrepnik N, Armas E, Swearingen CJ, DiFrancesco A, Tambiah JRS, Hood J, Hochberg MC. A novel Wnt pathway inhibitor, SM04690, for the treatment of moderate to severe osteoarthritis of the knee: results of a 24-week, randomized, controlled, phase 1 study. Osteoarthritis Cartilage. 2017;25:1598–606. 10.1016/j.joca.2017.07.006. 28711582

[16] Astrike-Davis EM, Coryell P, Loeser RF. Targeting cellular senescence as a novel treatment for osteoarthritis. Curr Opin Pharmacol. 2022;64. 10.1016/j.coph.2022.102213. PMC9167736 35447516

[17] Baechle JJ, Chen N, Makhijani P, Winer S, Furman D, Winer DA. Chronic inflammation and the hallmarks of aging. Mol Metab. 2023;74:101755. 10.1016/j.molmet.2023.101755. PMC10359950 37329949

[18] Kulkarni AS, Aleksic S, Berger DM, Sierra F, Kuchel GA, Barzilai N. Geroscience-guided repurposing of FDA-approved drugs to target aging: a proposed process and prioritization. Aging Cell. 2022;21. 10.1111/acel.13596. PMC9009114 35343051

[19] Leite CBG, Merkely G, Charles JF, Lattermann C. From inflammation to resolution: specialized pro-resolving mediators in posttraumatic osteoarthritis. Curr Osteoporos Rep. 2023;21:758–70. 10.1007/s11914-023-00817-3. 37615856

[20] Lim WB, Al-Dadah O. Conservative treatment of knee osteoarthritis: a review of the literature. World J Orthop. 2022;13:212–29. 10.5312/wjo.v13.i3.212. PMC8935331 35317254

[21] Ungvari Z, Fazekas-Pongor V, Csiszar A, Kunutsor SK. The multifaceted benefits of walking for healthy aging: from Blue Zones to molecular mechanisms. Geroscience. 2023;45:3211–39. 10.1007/s11357-023-00873-8. PMC10643563 37495893

[22] Jüni, P., Hari, R., Rutjes, A.W.S., Fischer, R., Silletta, M.G., Reichenbach, S., da Costa, B.R., 2015. Intra-articular corticosteroid for knee osteoarthritis. Cochrane Database Syst. Rev. 2015, CD005328. 10.1002/14651858.CD005328.pub3. PMC8884338 26490760

[23] Raynauld JP, Buckland-Wright C, Ward R, Choquette D, Haraoui B, Martel-Pelletier J, Uthman I, Khy V, Tremblay JL, Bertrand C, Pelletier JP. Safety and efficacy of long-term intraarticular steroid injections in osteoarthritis of the knee: a randomized, double-blind, placebo-controlled trial. Arthritis Rheum. 2003;48:370–7. 10.1002/art.10777. 12571845

[24] Bensa A, Previtali D, Sangiorgio A, Boffa A, Salerno M, Filardo G. PRP injections for the treatment of knee osteoarthritis: the improvement is clinically significant and influenced by platelet concentration: a meta-analysis of randomized controlled trials. Am J Sports Med. 2025;53:745–54. 10.1177/03635465241246524. PMC11874499 39751394

[25] Curtis A, Beswick A, Jenkins L, Whitehouse MR. Is there a role for autologous conditioned serum injections in osteoarthritis? A systematic review and meta-analysis of randomised controlled trials. Osteoarthritis Cartilage. 2024;32:1197–206. 10.1016/j.joca.2024.06.004. 38878817

[26] Cao M, Ou Z, Sheng R, Wang Q, Chen X, Zhang C, Dai G, Wang H, Li J, Zhang X, Gao Y, Shi L, Rui Y. Efficacy and safety of mesenchymal stem cells in knee osteoarthritis: a systematic review and meta-analysis of randomized controlled trials. Stem Cell Res Ther. 2025;16. 10.1186/s13287-025-04252-2. PMC11887158 40055739

[27] Gong J, Fairley J, Cicuttini FM, Hussain SM, Vashishtha R, Chou L, Wluka AE, Wang Y. Effect of stem cell injections on osteoarthritis-related structural outcomes: a systematic review. J Rheumatol. 2021;48:585–97. 10.3899/jrheum.200021. 33004537

[28] Heidari B. Knee osteoarthritis prevalence, risk factors, pathogenesis and features: Part I. Caspian J Intern Med. 2011;2:205–12. PMC3766936 24024017

[29] Makkar JKD, Warrier GD, Ghai BD, Chhabra M, Sarkar PKD, Goni VG, Kaur Khurana BJ. Effect of radiofrequency ablation of genicular nerves on the isokinetic muscle strength of knee joint in patients with osteoarthritis knee: a randomized double-blind sham controlled clinical trial. Pain Med. 2024;25:738–48. 10.1093/pm/pnae077. 39102829

[30] McAlindon TE, Driban JB, Henrotin Y, Hunter DJ, Jiang GL, Skou ST, Wang S, Schnitzer T. OARSI clinical trials recommendations: design, conduct, and reporting of clinical trials for knee osteoarthritis. Osteoarthritis Cartilage. 2015;23:747–60. 10.1016/j.joca.2015.03.005. 25952346

[31] Ro DH, Jang MJ, Koh J, Choi WS, Kim HC, Han HS, Choi JW. Mechanism of action of genicular artery embolization in a rabbit model of knee osteoarthritis. Eur Radiol. 2023;33:125–34. 10.1007/s00330-022-09006-9. 35932304

[32] Moyer RF, Birmingham TB, Bryant DM, Giffin JR, Marriott KA, Leitch KM. Valgus bracing for knee osteoarthritis: a meta-analysis of randomized trials. Arthritis Care Res (Hoboken). 2015;67:493–501. 10.1002/acr.22472. 25201520

[33] Hochberg MC, Martel-Pelletier J, Monfort J, Moller I, Castillo JR, Arden N, Berenbaum F, Blanco FJ, Conaghan PG, Domenech G, Henrotin Y, Pap T, Richette P, Sawitzke A, du Souich P, Pelletier JP. Combined chondroitin sulfate and glucosamine for painful knee osteoarthritis: a multicentre, randomised, double-blind, non-inferiority trial versus celecoxib. Ann Rheum Dis. 2016;75:37–44. 10.1136/annrheumdis-2014-206792. PMC4717399 25589511

[34] Veronese N, Koyanagi A, Stubbs B, Cooper C, Guglielmi G, Rizzoli R, Punzi L, Rogoli D, Caruso MG, Rotolo O, Notarnicola M, Al-Daghri N, Smith L, Reginster JY, Maggi S. Mediterranean diet and knee osteoarthritis outcomes: a longitudinal cohort study. Clin Nutr. 2019;38:2735–9. 10.1016/j.clnu.2018.11.032. PMC6451631 30553579

[35] Sanchez-Lopez E, Coras R, Torres A, Lane NE, Guma M. Synovial inflammation in osteoarthritis progression. Nat Rev Rheumatol. 2022;18:258–75. 10.1038/s41584-022-00749-9. PMC9050956 35165404

[36] Chappell AS, Desaiah D, Liu-Seifert H, Zhang S, Skljarevski V, Belenkov Y, Brown JP. A double-blind, randomized, placebo-controlled study of the efficacy and safety of duloxetine for the treatment of chronic pain due to osteoarthritis of the knee. Pain Pract. 2011;11:33–41. 10.1111/j.1533-2500.2010.00401.x. 20602715

[37] Kanjanapanang N, Madrid R, Lin P, Shilling M, Cooper A, Sen H, Thiyagarajan S, Chang KH, Luo H, Conger A, McCormick ZL, Ehsanian R. Effectiveness of genicular nerve radiofrequency ablation in osteoarthritis and post-surgical knee pain: systematic review. Pain Med. 2026;27:189–208. 10.1093/pm/pnaf115. 40855681

[38] DeRogatis M, Anis HK, Sodhi N, Ehiorobo JO, Chughtai M, Bhave A, Mont MA. Non-operative treatment options for knee osteoarthritis. Ann Transl Med. 2019;7(Suppl 7). 10.21037/atm.2019.06.68. PMC6828999 31728369

[39] Bender T, Bálint G, Prohászka Z, Géher P, Tefner IK. Evidence-based hydro- and balneotherapy in Hungary-a systematic review and meta-analysis. Int J Biometeorol. 2014;58:311–23. 10.1007/s00484-013-0667-6. PMC3955132 23677421

[40] Gálvez I, Torres-Piles S, Ortega-Rincón E. Balneotherapy, immune system, and stress response: a hormetic strategy? Int J Mol Sci. 2018;19. 10.3390/ijms19061687. PMC6032246 29882782

[41] Osuala U, Goh MH, Mansur A, Smirniotopoulos JB, Scott A, Vassell C, Yousefi B, Jain NK, Sag AA, Lax A, Park KW, Kheradi A, Sapoval M, Golzarian J, Habibollahi P, Ahmed O, Young S, Nezami N. Minimally invasive therapies for knee osteoarthritis. J Pers Med. 2024;14:970. 10.3390/jpm14090970. PMC11432885 39338224

[42] Duivenvoorden T, Brouwer RW, van Raaij TM, Verhagen AP, Verhaar JA, Bierma‐Zeinstra SM. Braces and orthoses for treating osteoarthritis of the knee. The Cochrane Database Systemat Rev 2015 Mar 16;2015(3):CD004020. 10.1002/14651858.CD004020.pub3. PMC7173742 25773267

[43] Buchbinder R, Osborne RH, Johnston RV, Pitt V. Self‐management education programmes for osteoarthritis. Cochrane Database Syst Rev. 2014. 10.1002/14651858.CD008963.pub2. PMC11104559 24425500

[44] Faber S, Brown A, Cottreau J. Safety of oral and topical nonsteroidal anti-inflammatory drugs. Orthop Nurs. 2024;43:234–7. 10.1097/NOR.0000000000001044. 39047276

[45] Krebs EE, Gravely A, Nugent S, Jensen AC, DeRonne B, Goldsmith ES, Kroenke K, Bair MJ, Noorbaloochi S. Effect of opioid vs nonopioid medications on pain-related function in patients with chronic back pain or hip or knee osteoarthritis pain: the SPACE randomized clinical trial. JAMA. 2018;319:872–82. 10.1001/jama.2018.0899. PMC5885909 29509867

[46] Sawitzke AD, Shi H, Finco MF, Dunlop DD, Bingham CO 3rd, Harris CL, Singer NG, Bradley JD, Silver D, Jackson CG, Lane NE, Oddis CV, Wolfe F, Lisse J, Furst DE, Reda DJ, Moskowitz RW, Williams HJ, Clegg DO. The effect of glucosamine and/or chondroitin sulfate on the progression of knee osteoarthritis: a report from the Glucosamine/Chondroitin Arthritis Intervention Trial. Arthritis Rheum. 2008;58:3183–91. 10.1002/art.23973. PMC2836125 18821708

[47] Feng J, Li Z, Tian L, Mu P, Hu Y, Xiong F, Ma X. Efficacy and safety of curcuminoids alone in alleviating pain and dysfunction for knee osteoarthritis: a systematic review and meta-analysis of randomized controlled trials. BMC Complement Med Ther. 2022;22. 10.1186/s12906-022-03740-9. PMC9580113 36261810

[48] Pereira TV, Jüni P, Saadat P, Xing D, Yao L, Bobos P, Agarwal A, Hincapié CA, da Costa BR. Viscosupplementation for knee osteoarthritis: systematic review and meta-analysis. BMJ. 2022;378. 10.1136/bmj-2022-069722. PMC9258606 36333100

[49] He WW, Kuang MJ, Zhao J, Sun L, Lu B, Wang Y, Ma JX, Ma XL. Efficacy and safety of intraarticular hyaluronic acid and corticosteroid for knee osteoarthritis: a meta-analysis. Int J Surg. 2017;39:95–103. 10.1016/j.ijsu.2017.01.087. 28137554

[50] Bennell KL, Paterson KL, Metcalf BR, Duong V, Eyles J, Kasza J, Wang Y, Cicuttini F, Buchbinder R, Forbes A, Harris A, Yu SP, Connell D, Linklater J, Wang BH, Oo WM, Hunter DJ. Effect of intra-articular platelet-rich plasma vs placebo injection on pain and medial tibial cartilage volume in patients with knee osteoarthritis: the RESTORE randomized clinical trial. JAMA. 2021;326:2021–30. 10.1001/jama.2021.19415. PMC8611484 34812863

[51] Raeissadat SA, Rayegani SM, Sohrabi MR, Jafarian N, Bahrami MN. Effectiveness of intra-articular autologous-conditioned serum injection in knee osteoarthritis: a meta-analysis study. Future Sci OA. 2021;7. 10.2144/fsoa-2021-0069. PMC8558851 34737891

[52] Epelboym Y, Mandell JC, Collins JE, Burch E, Shiang T, Killoran T, Macfarlane L, Guermazi A. Genicular artery embolization as a treatment for osteoarthritis-related knee pain: a systematic review and meta-analysis. Cardiovasc Intervent Radiol. 2023;46:760–9. 10.1007/s00270-023-03422-0. 36991094

[53] Little MW, O’Grady A, Briggs J, Gibson M, Speirs A, Al-Rekabi A, Yoong P, Ariyanayagam T, Davies N, Tayton E, Tavares S, MacGill S, McLaren C, Harrison R. Genicular artery embolisation in patients with osteoarthritis of the knee (GENESIS) using permanent microspheres: long-term results. Cardiovasc Intervent Radiol. 2024;47:1750–62. 10.1007/s00270-024-03752-7. PMC11621196 38819473

[54] Okuno Y, Korchi AM, Shinjo T, Kato S. Transcatheter arterial embolization as a treatment for medial knee pain in patients with mild to moderate osteoarthritis. Cardiovasc Intervent Radiol. 2015;38:336–43. 10.1007/s00270-014-0944-8. 24993956

[55] Taslakian B, Miller LE, Mabud TS, Macaulay W, Samuels J, Attur M, Alaia EF, Kijowski R, Hickey R, Sista AK. Genicular artery embolization for treatment of knee osteoarthritis pain: systematic review and meta-analysis. Osteoarthr Cartil Open. 2023;5. 10.1016/j.ocarto.2023.100342. PMC9971280 36865988

[56] Sapoval M, Querub C, Pereira H, Pellerin O, Boeken T, Di Gaeta A, Al Ahmar M, Lefevre-Colau MM, Nguyen C, Daste C, Lacroix M. Genicular artery embolization for knee osteoarthritis: results of the LipioJoint-1 trial. Diagn Interv Imaging. 2024;105:144–50. 10.1016/j.diii.2023.12.003. 38102013

[57] Milhem F, Takhman M, Elgendy MS, Zahra AA, Saife S, Saife S, Shehadeh W, Bdair M, Abu-Khazneh O, Hamdan Y, Ayaseh QZ, Hajjeh O, Younas A, Alya WA, Mohammad A, Zaitoun A. Genicular artery embolization for knee osteoarthritis: a systematic review of sham-controlled randomized trials. J Orthop. 2025;67:361–8. 10.1016/j.jor.2025.07.022. PMC12320538 40766107

[58] van Zadelhoff TA, van der Heijden RA, Bierma-Zeinstra SMA, Bos PK, Oei EHG. Long-term outcomes of genicular artery embolization for knee osteoarthritis: 12-month efficacy and secondary outcomes from a randomized sham-controlled clinical trial. Eur Radiol. 2026. 10.1007/s00330-026-12505-8. PMC13341897 42009866

